# The Influence of Plasticizers on the Response Characteristics of the Surfactant Sensor for Cationic Surfactant Determination in Disinfectants and Antiseptics

**DOI:** 10.3390/s21103535

**Published:** 2021-05-19

**Authors:** Nikola Sakač, Dubravka Madunić-Čačić, Maja Karnaš, Bojan Đurin, Ivan Kovač, Marija Jozanović

**Affiliations:** 1 Faculty of Geotechnical Engineering, University of Zagreb, 42000 Varaždin, Croatia; ivan.kovac@gfv.unizg.hr; 2 Saponia Chemical, Pharmaceutical and Foodstuff Industry, Inc., 31000 Osijek, Croatia; dubravka.madunic-cacic@saponia.hr; 3 Faculty of Agrobiotechnical Sciences Osijek, Josip Juraj Strossmayer University of Osijek, 31000 Osijek, Croatia; maja.karnas@fazos.hr; 4 Department of Civil Engineering, University North, 42000 Varaždin, Croatia; bdjurin@unin.hr; 5 Department of Chemistry, Josip Juraj Strossmayer University of Osijek, 31000 Osijek, Croatia

**Keywords:** plasticizer, sensor, potentiometry, antiseptic, cationic surfactant, disinfectant

## Abstract

Surfactant liquid-membrane type sensors are usually made of a PVC, ionophore and a plasticizer. Plasticizers soften the PVC. Due to their lipophilicity, they influence the ion exchange across the membrane, ionophore solubility, membrane resistance and, consequently, the analytical signal. We used the DMI-TPB as an ionophore, six different plasticizers [2-nitrophenyl-octyl-ether (P1), bis(2-ethylhexyl) phthalate (P2), bis(2-ethylhexyl) sebacate (P3), 2-nitrophenyl phenyl ether (P4), dibutyl phthalate (P5) and dibutyl sebacate (P6)] and a PVC to produce ionic surfactant sensors. Sensor formulation with P1 showed the best potentiometric response to four usually used cationic surfactant, with the lowest LOD, 7 × 10^−7^ M; and potentiometric titration curves with well-defined and sharp inflexion points. The sensor with P6 showed the lowest analytical performances. Surfactant sensor with P1 was selected for quantification of cationic surfactant in model solutions and commercial samples of disinfectants and antiseptics. It showed high accuracy and precision in all determinations, with recovery from 98.2 to 99.6, and good agreement with the results obtained with surfactant sensor used as a referent one, and a standard two-phase titration method. RDS values were lower than 0.5% for all determinations.

## 1. Introduction

Surface active agents or surfactants are widely used in the household and industry for washing, cleaning, disinfection and as emulsifiers. There are four main groups of surfactants: anionic, cationic, amphoteric and nonionic. Cationic surfactants are used in a broad spectra of commercial products as preservatives, disinfectants and antiseptics. In the time of the recent COVID-19 pandemic, global demands for disinfectants and antiseptic products are constantly rising. In this sense, there is a need to establish simple and inexpensive quality control platforms to quantify cationic surfactants in commercial product formulations. Since classical methods for the detection of lower surfactant concentrations [1] have many disadvantages, ion selective potentiometric sensors based on ion selective electrodes with a liquid membrane type [2,3,4] offer an elegant, affordable and reliable substitution. Potentiometric titrations with ion selective potentiometric sensors are usually used to quantify surfactants [5,6]. Liquid-membrane type sensing membranes are typically based on high molecular weight PVC mixed with low molecular weight plasticizer and an ionophore [5,7]. The typical weight ratio of high molecular weight PVC and a plasticizer is 1:2, with up to 1 wt % of added ionophore [8]. Higher amounts of plasticizer could be interfering with the measurement and decrease the sensitivity of the sensor. [9,10] PVC itself has a tendency to be brittle. For this reason, plasticizers offer mechanical stability [8]. Even though a plasticizer has a function to soften the matrix and make it more flexible, it also has an influence on the final potentiometric sensor response [11] since it influences the membrane polarity, resistance and ion mobility across the membrane. [8,12,13] Plasticizers are also important to protect the ionophore from leaching. As stated by Carey [9], the current focus is to find appropriate plasticizer that matches as closely as possible to the liphopilicity of the ionophore allowing the maximal amount of the ion-pair to be dissolved in the sensing membrane. Liphophilicity is related to the water penetration coefficient and with the water flux across the sensing membrane [14]. Carefully selected plasticizer influences not only the selectivity but also the lifetime of the sensor [15]. Additional requirements that the plasticizer should fulfill are: do not crystallize in the membrane, do not oxidize [16] and ensure the Nernstian response slope and lower the Ohmic resistance [8]. The focus of the plasticizer selection should be not only on the liphophilicity but also on their size and dielectric constant. Plasticizers with the higher dielectric constant are favorable. Measurements suggest that some plasticizers decrease in their dielectric constant when added to the PVC matrix [17]. For example, o-nitrophenyl octyl ether (o-NPOE) plasticizer in PVC membrane has a dielectric constant 14, which is much lower than in pure chemical form, where a dielectric constant is 21 [17]. For all these reasons, a high attention should be dedicated just to the selection of the appropriate plasticizer.

In a recent work, we conducted research to check the influence of different plasticizers on the properties of PVC-based potentiometric surfactant sensor for anionic sSDSurfactant detection. A dimethyldioctadecylammonium-tetraphenylborate (DDA-TPB) with new dibutyl phthalate plasticizer showed better properties in anionic surfactant detection, compared to the usually used o-NPOE plasticizer [13].

For this research, we used a 1,3-didecyl-2-methylimidazolium-tetraphenylborate (DMI-TPB) ionophore developed by our group. [18] In this paper, we investigated the influence of six different plasticizers implemented in PVC-based liquid membrane electrode with DMI-TPB as an ionophore, on the behavior of potentiometric surfactant sensors for cationic surfactant detection. The influence of plasticizers was investigated through potentiometric response characterization and potentiometric titration curves characterization. The best membrane formulation was selected and tested on commercial disinfectants and antiseptics containing cationic surfactants.

## 2. Materials and Methods

### 2.1. Reagents

Six different plasticizers were used to manufacture sensing membranes for surfactant sensors: 2-nitrophenyl-octyl-ether (P1), bis(2-ethylhexyl) phthalate (P2), bis(2-ethylhexyl) sebacate (P3), 2-nitrophenyl phenyl ether (P4), dibutyl phthalate (P5) and dibutyl sebacate (P6); all from Sigma Aldrich, Hamburg, Germany.

A high-molecular-weight PVC and tetrahydrofuran (THF), used for sensing membrane preparations, and 1,3-didecyl-2-methylimidazolium chloride (DMIC) and sodium tetraphenylborate (TPB), used for 1,3-didecyl-2-methylimidazolium-tetraphenylborate (DMI-TPB) membrane ionophore preparation, were all from Fluka, Buchs, Switzerland.

For potentiometric response measurements, four cationic surfactants were used: diisobutyl phenoxy ethoxy ethyl dimethyl benzyl ammonium chloride (Hyamine 1622), cetylpyridinium chloride (CPC), cetrimonium bromide (CTAB) and DMIC, all from Fluka, Buchs, Switzerland. Anionic surfactant sodium dodecyl sulfate (SDS) used as a titrant, was from Sigma Aldrich, Hamburg, Germany.

### 2.2. Membrane Preparation and Surfactant Sensors Fabrication

The membranes were prepared by dissolving previously synthesized DMI-TPB ionophore, a high-molecular-weight PVC (33%) and a corresponding plasticizer P1 to P6, (66%), in a small amount of THF. The final cocktail was transferred to the ultrasonic bath (Sonoplus Ultrasonic homogenizer with a horn sonicator HD 3100, from Bandelin, Germany) for sonication. The sensing mixture was transferred to 24 mm OD mold glass ring and left to dry. After two days, the THF evaporated and a thin plasticized layer was cut in a few 7 mm OD discs (sensor membranes) and stored dry for later use. The same procedure was used for each plasticizer separately. Phillips electrode body (Phillips IS-561, Glasbläserei Müller, Zürich, Switzerland) with 2M sodium chloride inner electrolyte was used to incorporate prepared sensor membranes and fabricate the surfactant sensor.

### 2.3. Response Measurements

Response measurements for cationic surfactants Hyamine 1622, CPC, CTAB and DMIC were performed for each sensing membrane formulation with corresponding plasticizer. Instrumentation for response measurements were Metrohm 780 pH meter and Metrohm 794 Basic Titrino with stirrer (Metrohm, Herisau, Switzerland). A silver/silver chloride reference electrode (Metrohm, Herisau, Switzerland) was paired with fabricated surfactant sensor. The measurements were performed in deionized water by adding corresponding increments of cationic surfactants (4 × 10^−2^ M to 4 × 10^−4^ M) to cover the broad concentration range. The increment interval for lower concentrations was from 120 s, and 60 s for higher cationic surfactant concentrations.

### 2.4. Potentiometric Titrations

Potentiometric titrations of cationic surfactants Hyamine 1622, CPC, CTAB and DMIC were performed with SDS as a titrant (*c* = 4 mM). A *Metrohm 808 Titrando* titrator with magnetic stirrer and *Tiamo 2.1* software, all from Metrohm, Herisau, Switzerland, connected to silver/silver chloride reference electrode (Metrohm, Herisau, Switzerland) paired with a corresponding surfactant sensor electrode, were used to perform potentiometric titrations. In titrations of the model solutions, the measuring volume was 25 mL (20 mL deionized water and 5 mL of corresponding cationic surfactant stock solution). The concentration of the cationic surfactant stock solutions used for titrations were 4 mM. The titrations of commercial pharmaceutical disinfectants and antiseptics provided in the local drug store, were performed by the same procedure for one selected membrane. pH was adjusted by adding NaOH (0.2 M) or HCl (*c* = 0.2 M) to the measuring solution.

## 3. Results and Discussion

### 3.1. Plasticizer Properties

To ensure the flexibility and elasticity of the PVC based liquid membranes, a plasticizer should be used. The focus of the plasticizer selection should be on their size, lipophilicity and dielectric constant. For this reason, a detailed table for six selected plasticizers (P1–P6) with their corresponding properties was presented in Table 1. Membrane properties included a molecular weight, calculated lipophilicity, a dielectric constant (ε), measured Ohmic resistance of the sensing membranes and their visual appearance.

Plasticizer lipophilicity is directly correlated with the water penetration coefficient and the water flux across the membrane [14]. Lipophilicity was calculated using the ALOGPS 2.1 software [13]. The most lipophilic plasticizer was bis (2-ethylhexyl) sebacate (P3) with lipophilicity 10.08, while 2-nitrophenyl phenyl ether (P4) showed the lowest lipophilicity, 3.39.

The Ohmic resistance (kΩ) of sensing membranes (230 µm thick) was measured by the loss of charge method [19] to check the influence of the plasticizer on the membrane resistance. According to measured resistance, the highest Ohmic resistance (74 MΩ) was measured for sensing membrane containing P6, while the lowest Ohmic resistance (55 MΩ) was measured for sensing membrane containing P1. Ohmic resistance of P2 (57 MΩ) was very close to P1 value. Plasticizer P1 had the highest dielectric constant value (21), while for P3 it was the lowest (3.89). Membranes had different visual appearances from transparent, to milky and opaque.

### 3.2. Potentiometric Response

**Figure 1:**
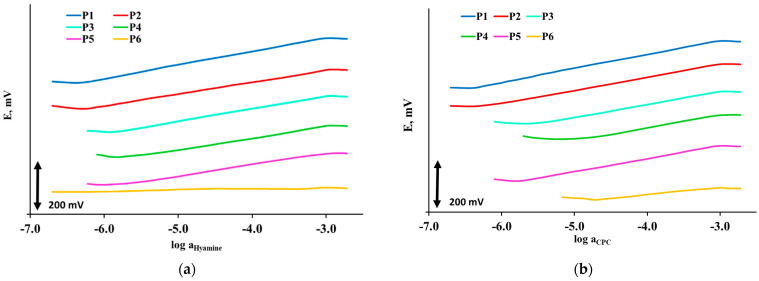

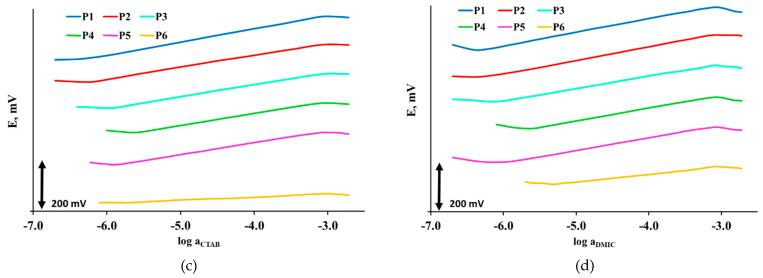
Response curves of the surfactant sensors toward (**a**) Hyamine 1622, (**b**) CPC, (**c**) CTAB and (**d**) DMIC in deionized water, for membranes containing six different plasticizers, from top to the bottom: 2-nitrophenyl-octyl-ether (P1), bis(2-ethylhexyl) phthalate (P2), bis (2-ethylhexyl) sebacate (P3), 2-nitrophenyl phenyl ether (P4), dibutyl phthalate (P5), dibutyl sebacate (P6).

Six plasticizers (P1–P6) were used to prepare six surfactant sensors. Each surfactant sensor was characterized by potentiometry. Potentiometric responses were measured for four monovalent cationic surfactants: Hyamine 1622, CPC, CTAB and DMIC. The corresponding response curves were presented in Figure 1. Most of the prepared sensing membranes showed good response characteristics. The lowest response to cationic surfactants, showed dibutyl sebacate, P6 plasticizer. For Hyamine 1622, P6 had no response. Statistical evaluations of the response curves were presented in Figure 1 to get more detailed information on response characteristics and select the most successful plasticizer for detection of cationic surfactants in model and real samples.

#### 3.2.1. Surfactant Sensors Responses to Hyamine 1622

Statistics of potentiometric response characteristics of surfactant sensors to Hyamine 1622 including calculated data on responses of PVC membrane formulations with six different plasticizers (P1–P6), given together with ±95% confidence limits were presented in Table 2.

Membrane formulation containing the P1 plasticizer showed a slope closest to the Nernstian, 58.78 ± 0.5, while the P6 plasticizer had no response. Correlation coefficients for all six plasticizers were within 0.99, within the useful linear concentration range. P1 had the highest useful concentration range from 3.2 × 10^−3^ to 3.9 × 10^−7^ M. Useful concentration range for plasticizer P2 was also very broad, ranging from 3.2 × 10^−3^ to 5.9 × 10^−7^, respectively. Useful concentration range for plasticizers P3-P5 was more narrow. The lowest limit of detection (LOD) was shown by P1 (2.1 × 10^−7^ M) and P2 (4.2 × 10^−7^ M), while other plasticizers had LOD in the range from 0.1 × 10^−6^ to 0.2 × 10^−6^ M.

#### 3.2.2. Surfactant Sensors Responses to CPC

Table 3 presented a statistics of potentiometric response characteristics of surfactant sensors to CPC including calculated data on responses of PVC membrane formulations with six different plasticizers (P1–P6), given together with ±95% confidence limits.

Membrane formulation containing the P1 plasticizer showed a slope closest to the Nernstian, 59.1 ± 0.5, while plasticizer 6 had the lowest slope value, 31.9 ± 0.2. Correlation coefficients for all six plasticizers were within 0.99, within the useful linear concentration range.

Plasticizer 1 had the highest useful concentration range from 3.2 × 10^−3^ to 3.9 × 10^−7^ M. Useful concentration range for plasticizer P2 was also very broad, being 3.2 × 10^−3^–5.9 × 10^−7^ M, respectively. Plasticizers P3 (3.2 × 10^−3^–3.5 × 10^−6^ M) and P5 (3.2 × 10^−3^–1.9 × 10^−6^ M) showed broader useful concentration ranges compared to P4 (3.2 × 10^−3^–1.3 × 10^−5^ M) and P6 (3.2 × 10^−3^–1.8 × 10^−5^ M). The lowest limit of detection (LOD) was obtained by P1 (2.1 × 10^−7^ M) and P2 (4.2 × 10^−7^ M), while other plasticizers had LOD in the range from 0.2 × 10^−5^ to 1.8 × 10^−6^ M.

#### 3.2.3. Surfactant Sensors Responses to CTAB

Table 4 presented the statistics of potentiometric response characteristics of surfactant sensors to CTAB including calculated data on responses of PVC membrane formulations with six different plasticizers (P1–P6), given together with ±95% confidence limits.

Membrane formulation containing P1 plasticizer showed a slope closest to the Nernstian, 59.2 ± 0.7, while plasticizer 6 had the lowest slope value, 14.1 ± 0.1. Correlation coefficients for all six plasticizers was within 0.99 (in linear range).

Plasticizer 1 had the highest useful concentration range from 3.2 × 10^−3^ to 3.9 × 10^−7^ M. Useful concentration range for plasticizer P2 was also very broad, 3.2 × 10^−3^ to 5.9 × 10^−7^ M, respectively.

Useful concentration range for plasticizers P3-P6 was more narrow. The lowest limit of detection (LOD) was shown by P1 (2.1 × 10^−7^ M) and P2 (4.2 × 10^−7^ M), while other plasticizers had LOD in the range from 1.9 × 10^−6^ to 0.1 × 10^−6^ M.

#### 3.2.4. Surfactant Sensors Responses to DMIC

Table 5 presented a statistics of potentiometric response characteristics of P1–P6 surfactant sensors on DMIC, including calculated data on responses of PVC membrane formulations with six different plasticizers (P1–P6), given together with ±95% confidence limits.

Membrane formulations containing P1 and P2 plasticizers showed a slope closest to the Nernstian, 59.0 ± 0.6 and 59.0 ± 0.7, respectively. P6 plasticizer, surprisingly, had the slope value 55.8 ± 0.4, making the membrane formulation with P6 possible to detect small changes in DMIC concentration. Correlation coefficients for all six plasticizers was within 0.999 (in linear range). Plasticizers P1 to P3 showed the widest useful concentration range from 4.8 × 10^−4^ to 7.7 (7.9) × 10^−7^ M. The lowest LOD was shown for P1, P2 and P3 (7 × 10^−7^ M).

Membrane formulations containing P1 and P2 plasticizers had a slope closest to the Nernstian, 59.6 ± 0.7 (P2) and 58.4 ± 0.6 (P1), while plasticizer P6 showed the lowest slope value, 32.9 ± 0.4. Correlation coefficients for all six plasticizers were within 0.99 (in linear range). Plasticizer P1 showed the highest useful concentration range from 33.2 × 10^−3^ to 3.9 × 10^−7^ M. Useful concentration ranges for plasticizers P2 and P3 were also very broad. The lowest limit of detection (LOD) was shown by P1 (2.1 × 10^−7^ M). Plasticizers P2 (4.2 × 10^−7^ M) and P3 (5.7 × 10^−7^ M) also showed low LODs, while other plasticizers had LODs in the range from 0.1 × 10^−6^ to 2.9 × 10^−6^ M.

### 3.3. Potentiometric Titrations

**Figure 2:**
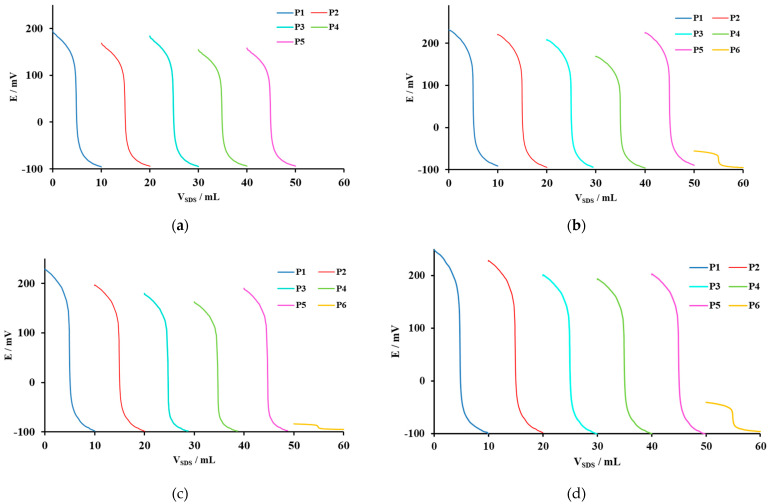
Potentiometric titration curves for (**a**) Hyamine 1622, (**b**) CPC, (**c**) CTAB and (**d**) DMIC, using NaSDS as a titrant (*c* = 4mM) and DMI-TPB sensors prepared with 6 different plasticizers: 2-nitrophenyl-octyl-ether (P1), bis(2-ethylhexyl) phthalate (P2), bis (2-ethylhexyl) sebacate (P3), 2-nitrophenyl phenyl ether (P4), dibutyl phthalate (P5) and dibutyl sebacate (P6). Some curves are displaced laterally and/or vertically, for clarity.

The behavior of the surfactant sensors was investigated on titrations of model solution for selected cationic surfactants with anionic surfactant SDS as a titrant. Cationic surfactants used in potentiometric titrations with SDS (*c* = 4 mM) were Hyamine 1622, CPC, CTAB and DMIC. All six surfactant sensor formulations were tested by potentiometric titration of each cationic surfactant (Figure 2).

The titration curves showed well-defined and sharp inflexion points (Figure 2), except for titration of Hyamine 1622 in membrane formulation with plasticizer P6. This was expected, since the potentiometric sensor responses of plasticizer P6 were low in most membrane formulations (and no signal was obtained for Hyamine 1622). However, the end-point could still be easily calculated by the 1st derivative, with a sharp end-point peak. For the sake of clarity, first derivative curves were not plotted in Figure 2, but their peak values were included in Table 6. Detailed data on titration curve parameters were presented in Table 6.

The titration curves of Hyamine 1622 titrations with NaSDS (*c* = 4 mM), using DMI-TPB sensors containing six different plasticizers, showed the highest potential change for P1 plasticizer, up to 290.1 mV, and the use of sensor containing P6 had no signal (Table 6). This was expected since P6 gave no response signal to Hamine 1622. The highest value of d*E*/d*V* change at the end-point was shown by the sensor with P1.

The titration curves of CPC titrations with SDS (*c* = 4 mM), showed the highest potential change for P1 plasticizer, up to 321.7 mV, while the P6 had the lowest potential change, up to 39.4 mV (Table 6). The highest value of dE/dV change at the end-point was shown by P1.

The titration curves of CTAB titration with SDS (*c* = 4 mM), using DMI-TPB sensors containing six different plasticizers, showed the highest potential change for P1 plasticizer, up to 325.7 mV (the highest value of d*E*/d*V* change at the end-point), and the use of sensor containing P6 had the lowest potential change, up to 11.6 mV (Table 6).

The titration curves of DMIC titration with SDS (*c* = 4 mM) showed the highest potential change for P1 plasticizer, up to 357.6 mV (the highest value of d*E*/d*V* change at the end-point), and the use of sensor containing P6 exhibited the lowest potential change, up to 54.6 mV (Table 6).

For potentiometric response measurements of cationic surfactants, sensing membrane formulations containing plasticizers P1 and P2 had excellent response characteristics. P1 showed the lowest LOD values for all investigated cationic surfactants. Sensing membrane formulations containing P1 to P5 showed well-defined titration curves and sharp inflexion points for titrations of all four investigated cationic surfactants. Plasticizer P1 had the best titration performances. Membrane formulation with plasticizer P6 showed titration curves with low signal change, but still sharp inflexion, except for Hyamine 1622 where no signal was obtained.

The role of the plasticizer is to soften the rigid PVC membrane. The investigated sensing membrane formulations had a 66% of plasticizer and 33% of PVC. Higher amounts of plasticizer are interfering to the measurement and could have a negative impact on the sensitivity. Plasticizer is a solvent for the ionophore and prevents leaching. All selected plasticizers were polymeric plasticizers and as such were not susceptible to migration and extraction. Higher amounts of plasticizer could also act as a solvent of lipophilic impurities resulting in shorter lifetime and a signal loss.

Even though the lipophilicity of 2-nitrophenyl-octyl-ether (P1) was not the highest among selected plasticizers (Table 1), in combination with the highest dielectric constant (21), P1 showed the best sensor performances.

The aim of this research was to investigate the influence of plasticizers (in sensor membrane formulations) on potentiometric detection of cationic surfactants in commercial products, such as disinfectants and antiseptics where cationic surfactant concentrations are high; from measuring results it can be concluded that sensing membrane formulation with P1 plasticizer is the best for cationic surfactant detection at concentration range of cationic surfactants usually expected in commercial products. It is also important to note that the price of DMIC is much higher than the others, and it is usually not used in commercial products. On the other side, CPC (as a second best) is the most frequently used disinfectant and anti-bactericidal agent, especially in current situation of global COVID-19 pandemic. For this reason, for further investigation of cationic surfactants in commercial samples of disinfectants and antiseptics we selected the sensing membrane formulation with P1 plasticizer.

### 3.4. Titration of Commercial Disinfectants and Antiseptics

The sensing membrane formulation with P1 plasticizer and a DMI-TPB ionophore was employed for end-point detection of cationic surfactants in potentiometric titrations of commercial samples of disinfectants and antiseptics. To observe the behavior of selected P1 membrane in the complex matrix, the standard addition method was carried out. The known amount of CPC was added in the commercial sample solution and titrated with SDS (*c* = 4 mM) as a titrant (Table 7). Three samples of commercial disinfectants and antiseptics purchased at the local drugstore were used. The known addition method, at two concentration level of added CPC, showed high recovery rates from 98.2% to 99.4%, for all concentrations of added CPC.

Six samples of commercial disinfectants and antiseptics containing cationic surfactants were used for potentiometric titrations with selected DMI-TPB sensor containing plasticizer P1 as an end-point indicator, and SDS (*c* = 4 mM) as a titrant, at the 95% confidence level. The RDS values for DMI-TPB sensor with P1 were lower than 0.5% for all measured samples. No significant differences were observed between the means of the selected surfactant sensor and a recently published potentiometric surfactant sensor for cationic surfactants [20] and results obtained by the colorimetric two-phase titration method which is a standard method [21] (Table 8). A two-phase titration method is a colorimetric method. As an end-point indicator solvatochromic dyes, such as dimidium bromide or disulphine blue mixed indicator, are used. The end-point is reached when the layer with organic solvent (chloroform) changes color. For this reason, it is hard to perform, since it requires experienced analyst and uses toxic organic solvents. Selected DMI-TPB sensor with P1 showed high accuracy and precision in all determinations, with high recoveries and a good agreement with referent methods.

## 4. Conclusions

DMI-TPB ionophore was used with six selected plasticizers 2-nitrophenyl-octyl-ether (P1), bis(2-ethylhexyl) phthalate (P2), bis(2-ethylhexyl) sebacate (P3), 2-nitrophenyl phenyl ether (P4), dibutyl phthalate (P5) and dibutyl sebacate (P6)) and a PVC to produce surfactant sensors.

Potentiometric responses of four usually used cationic surfactants (Hyamine 1622, CPC, CTAB and DMIC) showed the best performances for sensors with P1 and P2. Sensor formulation with P1 showed the lowest LOD, 7 × 10^−7^ M. Sensor formulation with P6 had the lowest analytical performances in all investigations.

Potentiometric titration had a well-defined titration curves and sharp inflexion points for titrations of all investigated cationic surfactants, where P1 plasticizer showed outstanding titration performances, fast response time, high signal change and signal stability.

Surfactant sensor with P1 was selected for quantification of the cationic surfactant in commercial samples of disinfectants and antiseptics and showed good agreement with the referent surfactant sensor and a referent two-phase titration method. The known addition method was used to check the matrix influence and showed good recoveries (98.2% to 99.6%).

Since the selected formulation of PVC-based surfactant sensor with DMI-TPB ionosphere and 2-nitrophenyl-octyl-ether (P1) showed outstanding characteristics, there is a high potential to use the developed sensor for fast and reliable analysis and quality control of cationic surfactants in industry.

## Figures and Tables

**Table 1:** Plasticizers and sensor membranes properties.

Plasticizer Name	Abbreviation	Mr g/mol	Lipophilicity *	Dielectric Constant (ε) **	Resistance (MΩ)	Membrane Visual Appearance
2-nitrophenyl-octyl-ether	P1	251.32	5.80	21	55	opaque
bis(2-ethylhexyl) phthalate	P2	390.56	7.60	5.17	57	milky
bis(2-ethylhexyl) sebacate	P3	426.67	10.08	3.89	62	milky
2-nitrophenyl phenyl ether	P4	215.20	3.39	9.8	61	transparent
dibutyl phthalate	P5	278.34	4.79	6.43	59	opaque
dibutyl sebacate	P6	314.46	6.30	4.6	74	opaque

* calculated using the *ALOGPS 2.1* online program. ** [17].

**Table 2:** Statistics of the response characteristics of the P1–P6 surfactant sensors to Hyamine, given together with ±95% confidence limits.

Parameters	Surfactant Sensor with Plasticizer					
	P1	P2	P3	P4	P5	P6
Slope, mV/decade of activity	58.78 ± 0.6	53.61 ± 1.1	56.61 ± 0.5	50.6 ± 1.0	50.67 ± 1.1	-/-
Standard error	0.4	0.6	0.3	0.7	0.7	-/-
Correlation coefficient, R^2^	0.9935	0.9993	0.9992	0.9964	0.9965	-/-
Detection limit, M	2.1 × 10^−7^	4.2 × 10^−7^	0.1 x 10^−6^	0.2 × 10^−6^	0.1 x 10^−6^	-/-
Useful conc. Range, M	3.2 × 10^−3^–3.9 × 10^−7^	3.2 × 10^−3^–5.9 x 10^−7^	3.2 × 10^−3^–1.2 × 10^−6^	3.2 × 10^−3^–1.6 × 10^−6^	3.2 × 10^−3^–1.2 × 10^−6^	-/-

**Table 3:** Statistics of the response characteristics of the P1–P6 surfactant sensors on CPC, given together with ±95% confidence limits.

Parameters	Surfactant Sensor with Plasticizer					
	P1	P2	P3	P4	P5	P6
Slope, mV/decade of activity	59.1 ± 0.5	57.8 ± 0.6	55.8 ± 0.4	55.9 ± 1.3	57.47 ± 0.6	31.9 ± 0.2
Standard error	0.4	0.4	0.4	0.2	0.3	0.5
Correlation coefficient, R^2^	0.9945	0.9997	0.9986	0.9968	0.9996	0.9955
Detection limit, M	2.1 × 10^−7^	4.2 × 10^−7^	1.8 × 10^−6^	0.1 × 10^−5^	0.2 × 10^−6^	0.2 × 10^−5^
Useful conc. Range, M	3.2 × 10^−3^–3.9 × 10^−7^	3.2 × 10^−3^–5.9 × 10^−7^	3.2 × 10^−3^–3.5 × 10^−6^	3.2 × 10^−3^–1.3 × 10^−5^	3.2 × 10^−3^–1.9 × 10^−6^	3.2 × 10^−3^–1.8 × 10^−5^

**Table 4:** Statistics of the response characteristics of the P1–P6 surfactant sensors on CTAB, given together with ±95% confidence limits.

Parameters	Surfactant Sensor with Plasticizer					
	P1	P2	P3	P4	P5	P6
Slope, mV/decade of activity	59.2 ± 0.7	53.5 ± 0.5	52.8 ± 0.5	53.01 ± 0.6	52.9 ± 0.5	14.1 ± 0.1
Standard error	0.7	0.7	0.4	0.3	0.5	0.044
Correlation coefficient, R^2^	0.9996	0.9991	0.9992	0.9984	0.9999	0.9986
Detection limit, M	2.1 × 10^−7^	4.2 × 10^−7^	0.1 × 10^−6^	0.9 × 10^−6^	0.2 × 10^−6^	1.9 × 10^−6^
Useful conc. Range, M	3.2 × 10^−3^–3.9 × 10^−7^	3.2 × 10^−3^–5.9 × 10^−7^	3.2 × 10^−3^–1.2 × 10^−6^	3.2 × 10^−3^–2.6 × 10^−6^	3.2 × 10^−3^–1.6 × 10^−6^	3.2 × 10^−3^–3.9 × 10^−6^

**Table 5:** Statistics of the response characteristics of the P1–P6 surfactant sensors to DMIC, given together with ±95% confidence limits.

Parameters	Surfactant Sensor with plasticizer					
	P1	P2	P3	P4	P5	P6
Slope, mV/decade of activity	58.4 ± 0.6	59.6 ± 0.7	54.6 ± 0.7	55.1 ± 0.7	55.9 ± 0.5	32.9 ± 0.4
Standard error	0.2	0.4	0.2	0.4	0.7	0.6
Correlation coefficient, R^2^	0.9994	0.9997	0.9998	0.9997	0.9999	0.9994
Detection limit, M	2.1 × 10^−7^	4.2 × 10^−7^	5.7 × 10^−7^	0.9 × 10^−6^	0.1 × 10^−6^	2.9 × 10^−6^
Useful conc. Range, M	3.2 × 10^−3^–3.9 × 10^−7^	3.2 × 10^−3^–5.9 × 10^−7^	3.2 × 10^−3^–7.9 × 10^−7^	3.2 × 10^−3^–2.6 × 10^−6^	3.2 × 10^−3^–1.2 × 10^−6^	3.2 × 10^−3^–4.9 × 10^−6^

**Table 6:** Titration data and statistics for (a) Hyamine 1622, (b) CPC, (c) CTAB and (d) DMIC titration with SDS (c = 4 mM) using DMI-TPB sensors prepared with six different plasticisers (P1–P6). Δ*E*, mV is the difference between the end and the start potential; d*E*/d*V* is the value of first derivative in the end-point. The statistics were based on five independent measurements.

		DMI-TPB Sensor Plasticizer Type					
		P1	P2	P3	P4	P5	P6
Hyamine 1622 with SDS	Δ*E*, m*V*	290.1	263.4	278.2	248.6	252.4	-/-
	d*E*/d*V*	61.5	59.1	43:5	43.1	55.3	-/-
	SDS consumption in EP, mL	4.83	4.83	4.79	5.07	4.79	-/-
	SD of EP	0.013	0.023	0.018	0.054	0.018	-/-
CPC with SDS	Δ*E*, m*V*	321.7	314.9	304.0	263.8	313.3	39.4
	d*E*/d*V*	69.4	67.7	46.4	45.0	62.2	9.6
	SDS consumption in EP, mL	4.78	4.76	4.69	4.79	4.72	4.86
	SD	0.016	0.018	0.016	0.059	0.019	0.035
CTAB with SDS	Δ*E*, m*V*	325.7	294.7	276.2	260.2	286.1	11.6
	d*E*/d*V*	71.5	67.9	54.8	56.9	65.2	13.9
	SDS consumption in EP, mL	4.97	4.92	4.92	4.91	4.94	4.96
	SD	0.024	0.015	0.037	0.035	0.038	0.027
DMIC with SDS	Δ*E*, m*V*	357.6	325.2	300.3	292.5	303.2	54.6
	d*E*/d*V*	66.6	61.4	52.4	46.2	59.1	33.8
	SDS consumption in EP, mL	4.73	4.70	4.69	4.75	4.71	4.96
	SD	0.027	0.015	0.014	0.026	0.015	0.025

**Table 7:** Titration of cationic surfactants in commercial samples of disinfectants and antiseptics with the known addition method using CPC addition, and selected DMI-TPB sensor containing plasticizer P1 as an end-point indicator. SDS (*c* = 4 mM) was used as a titrant.

Commercial Sample	CPC Taken/Mol	CPC Found/Mol
1	1 × 10^−5^	0.989 × 10^−5^
	1 × 10^−4^	0.982 × 10^−5^
2	1 × 10^−5^	0.996 × 10^−5^
	1 × 10^−4^	0.992 × 10^−5^
3	1 × 10^−5^	0.989 × 10^−5^
	1 × 10^−4^	0.991 × 10^−5^

**Table 8:** The results of potentiometric titrations of cationic surfactants in commercial disinfectants and antiseptics samples using SDS (*c* = 4 mM) as a titrant and DMI-TPB sensor containing plasticizer P5 as an indicator, in comparison with published DMI-TPB sensor containing P1.

Product	CATIONIC SURFACTANT CONTENT *				
	DMI-TPB Sensor with P1		Surfactant Sensor **		Two-Phase Titration
	%	RSD (%)	%	RSD (%)	%
A	4.38	0.36	4.32	0.41	4.33
B	5.26	0.38	5.13	0.62	5.15
C	4.70	0.31	4.76	0.42	4.71
D	4.80	0.35	4.72	0.48	4.77
E	0.06	0.26	0.07	0.35	0.07
F	0.15	0.45	0.15	0.25	0.16

* average of 5 determinations; ** sensor developed in [20].

## Data Availability

The data presented in this study are available on request.
